# The Causal Effect of Urate Level on Female Infertility: A Mendelian Randomization Study

**DOI:** 10.3390/metabo14100516

**Published:** 2024-09-25

**Authors:** Jiawei Sun, Ting Shen, Yining Guan, Yixin Jiang, Xiaoling Xu

**Affiliations:** 1 Shulan International Medical College, Zhejiang Shuren University, Hangzhou 310052, China; 2 School of Medicine, Zhejiang University, No. 866 Yuhantang Road, Hangzhou 310058, China

**Keywords:** urate, female infertility, mendelian randomization, causal inference

## Abstract

Background/Objective: This study aimed to investigate the causal relationship between urate level and female infertility using Mendelian randomization (MR) analysis. Methods: To identify instrumental variables, we selected independent genetic loci associated with serum urate levels in individuals of European ancestry, utilizing data from large-scale genome-wide association studies (GWAS). The GWAS dataset included information on serum urate levels from 288,649 CKDGen participants. Female infertility data, including different etiologic classifications, consisted of 13,142 female infertility patients and 107,564 controls. We employed four MR methods, namely inverse variance weighted (IVW), MR-Egger, weighted median, and weighted model, to investigate the causal relationship between urate levels and female infertility. The Cochran Q-test was used to assess heterogeneity among single nucleotide polymorphisms (SNPs), and the MR-Egger intercept test was employed to evaluate the presence of horizontal pleiotropy. Additionally, a “leave-one-out” sensitivity analysis was conducted to examine the influence of individual SNPs on the MR study. Results: The IVW analysis demonstrated that elevated serum urate levels increased the risk of female infertility (odds ratio [OR] = 1.18, 95% confidence interval [CI]: 1.07–1.33). Furthermore, serum urate levels were found to be associated with infertility due to cervical, vaginal, or other unknown causes (OR = 1.16, 95% CI: 1.06–1.26), also confirmed by other methods. Heterogeneity among instrumental variables was assessed using Cochran’s Q-test (*p* < 0.05), so a random-effects IVW approach was employed in the effects model. The MR-Egger intercept test indicated no presence of horizontal pleiotropy. A “leave-one-out” sensitivity analysis was conducted, demonstrating that no individual SNP had a substantial impact on the overall findings. Conclusions: In the European population, the urate level is significantly and causally associated with an increased risk of female infertility.

## 1. Introduction

Failure to achieve pregnancy after 12 months of unprotected intercourse is clinically defined as infertility [1]. Infertility represents a significant global health concern, particularly among couples in their childbearing years [2]. Infertility affects approximately 10–15% of couples in the reproductive age group, and the World Health Organization (WHO) predicts that it will emerge as one of the three major challenges to human health, alongside cancer and cardiovascular disease [3,4]. It serves as a critical determinant of population well-being and family satisfaction, and it serves as a pivotal indicator for assessing reproductive health standards, medical services, and economic and cultural development, as well as social progress at various levels within a country or region [5]. The ramifications of infertility extend beyond the individual and societal levels, adversely influencing fertility rates across multiple nations. Common causes of female infertility encompass ovulatory dysfunction, uterine adhesions, and tubal disorders [6,7]. Tubal infertility is a prevalent issue, contributing to approximately 20–40% of infertility cases and standing as the primary cause of female infertility [6]. Following closely behind tubal factors, ovulatory disorders account for around 10–30% of female infertility cases. Additionally, endometriosis is found in conjunction with infertility in 30–50% of patients [8]. In cases where all tests have been completed and results are normal, yet the cause of infertility remains unknown, it is commonly referred to as an unspecified origin. Despite the label of “unspecified”, it does not imply the absence of an underlying cause, but rather signifies that the specific cause has not yet been identified, often involving immunologic factors and altered uterine tolerance [9].

Urate serves as the principal metabolite resulting from the oxidation of purines within the human body [10]. The impact of serum urate levels on physiological processes varies depending on its concentration [11]. Optimal levels of serum urate have been shown to enhance the antioxidant capacity of the human body, thereby exerting inhibitory effects on nitric oxide production, safeguarding endothelial function, and scavenging reactive oxygen species [12,13]. Extensive evidence has established a significant association between elevated serum urate levels and renal and cardiovascular diseases [14,15]. However, limited research has been conducted regarding the interplay between urate and the female reproductive system. Elevated uric acid levels have been found to be associated with an increased susceptibility to anovulatory disease [16]. Urate, a purine derivative, has the potential to impede oocyte maturation due to the inhibitory effects of purines [17,18]. Heightened levels of urate can induce oxidative stress, resulting in detrimental effects on the female reproductive system [19], abnormal lipid metabolism [20], and systemic aseptic inflammation [21]. Numerous studies have demonstrated that elevated serum urate levels exacerbate the metabolic abnormalities observed in polycystic ovary syndrome [21,22,23]. Furthermore, patients with polycystic ovary syndrome and elevated serum urate levels face an augmented risk of hyperandrogenism, which, in turn, leads to heightened levels of follicular atresia in women, ultimately impairing ovulation [24]. In cases where elevated urate levels trigger inflammation, the interleukin-1β response may induce ovulation while inhibiting endometrial chemotaxis, potentially influencing female embryo implantation and resulting in female infertility [25]. Although certain epidemiological studies have indicated elevated serum urate levels in patients with polycystic ovary syndrome (PCOS) [26,27] and endometriosis [21,28], direct investigations exploring the correlation between high serum urate levels and female infertility remain scarce. Furthermore, no studies have employed a Mendelian randomization approach to evaluate the causal relationship between elevated urate levels and female infertility.

One study utilized data from the National Health and Nutrition Examination Survey (NHANES) database to analyze and conclude that higher serum urate levels increase the risk of infertility in women [29]; however, this cross-sectional study, which was not able to show a causal relationship between serum urate levels and female infertility, and the causal relationship between exposures and disease outcomes, is susceptible to confounding factors and reverse causality effects, leading to a bias in the inference of cause and effect in the bias in causal inference. To reduce the effects of acquired confounders and reverse causation, we introduced a Mendelian randomization (MR) approach [30,31]. In this MR study, we explored the association of selected exposures predicted by genetic variation with the corresponding outcomes. Since an individual’s genetic variation cannot be modified after conception, the use of genetic variation as an instrumental variable (IV) can greatly improve the reliability of the results. Furthermore, based on the availability of publicly available genetic data, this method can be widely applied.

In this MR analysis, we aimed to demonstrate whether there is a causal relationship between female infertility and serum urate levels.

## 2. Methods

### 2.1. Study Design Overview

**Figure 1:**
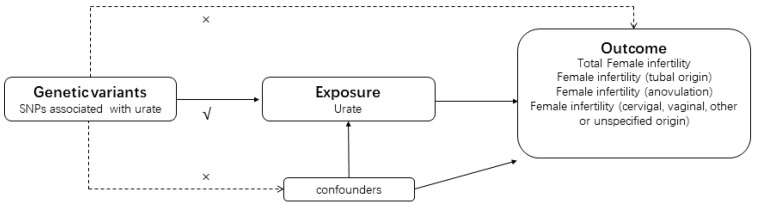
The design of this bidirectional MR study. The ‘×’ means that genetic variants are not associated with confounders or cannot be directly involved in outcome but via the exposure pathway. The ‘√’ means that genetic variants are highly correlated with exposure. Solid paths are significant; dashed paths should not exist in the MR study. SNP, single nucleotide polymorphism.

To investigate the causal relationship between urate levels and infertility, we conducted a bidirectional MR study employing genetic variation as IVs [32]. The study design, illustrated in Figure 1, adhered to rigorous criteria outlined as follows: Firstly, genetic variants were meticulously selected to exhibit a high degree of correlation with urate levels. Secondly, these genetic variations were ensured to be unrelated to confounding factors. Then the genetic variations were confirmed to exert their influence on infertility solely through the exposure pathway, without direct involvement in the outcome. To achieve robust results, we leveraged pooled statistical data from a recent meta-analysis of genome-wide association studies (GWAS) focused on urate and infertility.

### 2.2. Exposure Data Sources and IV Selection

We obtained summary statistics on serum urate levels from published meta-analyses of genome-wide association studies (GWAS) involving a cohort of approximately 288,649 individuals of European ancestry from the CKDGen consortium [33] and replication of study results using a female-specific exact GWAS summary statistics [34]. The GWAS for body mass index includes 461,460 European populations and 9,851,867 SNPs. The characteristics of the study population can be found in Supplementary Table S1. Using R software 4.3.1, we integrated the screened single nucleotide polymorphisms (SNPs) that showed genome-wide significance. Variants associated with serum urate levels of genome-wide significance were identified using a threshold of *p* < 5 × 10^−8^ as the primary screening criterion. Subsequently, we excluded SNPs in linkage disequilibrium (SNPs with r^2^ < 0.001 and a physical distance exceeding 10,000 kb between every two genes) and selected SNPs with the smallest *p* values. Furthermore, we assessed the strength of the IVs by computing the R^2^ and F statistic, considering the sample size of the exposure dataset, the number of IVs, and the genetic variance [33]. Finally, we conducted an analysis in the Phenoscanner database to search for exposure-related SNPs and their proxies, aiming to identify SNPs associated with potential confounders (*p* < 1 × 10^−5^). To avoid potential pleiotropic effects, these SNPs were manually excluded. Supplementary Tables S2 and S3 provide detailed information on the identified urate-associated SNPs.

### 2.3. Outcome Data Sources and IV Selection

The present study utilized population-based meta-analysis data from 2023 to investigate female infertility and its various etiologies. The dataset included 13,142 female infertility patients and 107,564 controls. The diagnostic criteria for infertility were established according to the ICD-10 classifications. To access all the data used in this study, please refer to the FinnGen Consortium version R9, available for download at https://r9.finngen.fi/ (accessed on 19 July 2024) [35]. For a comprehensive overview of the findings, please consult Supplementary Table S1, which provides detailed information regarding the results.

### 2.4. MR Analysis

All statistical analyses were conducted using a “TwoSampleMR” package in R software version 4.1.2 [36]. To estimate the causal effect between urate level and infertility in our study, we employed four distinct methods: inverse variance weighted (IVW) [37], MR-Egger [38], weighted median [39], and weighted model. The IVW method, known for its classical approach in MR analysis, served as our primary method. It involves assigning weights based on the inverse variance of each instrumental variable, ensuring the validity of all instrumental variables. Heterogeneity among individual SNP estimates was evaluated using Cochran’s Q-test, where a statistically significant result indicated significant heterogeneity in the analysis [40]. Additionally, we employed the MR-Egger intercept test to assess the horizontal pleiotropy of SNPs. A statistically significant intercept term in the MR-Egger intercept test analysis would suggest significant horizontal pleiotropy in the MR analysis. Compared with MR-Egger, the MR-PRESSO Global-test has higher accuracy and is useful in identifying horizontal pleiotropy and outlier; a *p* value of Global test < 0.05 indicates the presence of horizontal pleiotropy [41]. We performed the MR Steiger test to estimate the potential reverse causal impact of exposure on outcome. Furthermore, we performed “leave-one-out” sensitivity analyses, systematically removing a single SNP at a time, to evaluate the influence of each variant on the association between the exposure and outcome variables. The statistical tests for the MR analyses of female infertility and its three different types were considered statistically significant at *p* < 0.0125 ([*p* < 0 .05]/4 outcome measures). In this study, ORs were interpreted as odds for outcome per unit increase in exposure. For levels of urate, the unit was mg/dL.

As an extension of two-sample MR, multivariate MR can estimate the causal impact of various risk factors together on female infertility by including all exposures in the same model. To demonstrate that urate level characteristics are independent of the direct effect of body mass index on female infertility risk that is not mediated through other exposures. Significantly associated SNPs were extracted and then combined with existing exposure instrumental variables. After excluding these duplicated SNPs, results from exposure and outcome effects and corresponding standard errors were obtained for each SNP from the exposure and results. The IVW method was used to infer causality in multivariate MR analyses.

### 2.5. Ethical Considerations and Informed Consent

This study utilized publicly available data from GWAS studies that had received approval from relevant institutional review boards and obtained informed consent from participants, caregivers, legal guardians, or other representatives.

## 3. Results

### 3.1. Association between Serum Urate Levels and Female Infertility

After querying the online platform SNiPA (https://snipa.helmholtz-muenchen.de/ (accessed on 19 July 2024)), we excluded five SNPs for which proxies were not available. Using the Phenoscanner database we ensured that all SNPs and sexes were uncorrelated. Consequently, 93 independent genetic variants in linkage disequilibrium were utilized as IVs for urate (Table S3). This study was not affected by weak instrumental variables, as all F-statistics of the urate IVs exceeded the threshold of 10, reducing bias in the estimation of IVs.

We detected heterogeneity in the association between female infertility and the selected urate IVs using Cochran’s Q-test (*p* < 0.05) (Table 1). Therefore, we employed an inverse variance weighting (IVW) approach in the random effects model. In the IVW model, elevated serum urate levels were causally associated with overall female infertility [odds ratio (OR) = 1.14, 95% confidence interval (CI) = 1.04, 1.25]. The results obtained from the MR-Egger, weighted model, and weighted median models were consistent with the IVW model (Table 1, Figures S1 and S2A). The MR-Egger analyses did not indicate any directional pleiotropy for the IVs (*p* intercept = 0.33), the MR-PRESSO test also shows no potential horizontal pleiotropy (*p* > 0.05) (Table 1), and the funnel plot demonstrated no evidence of bias in the study (Supplementary Figure S3A), The MR Steiger test indicated that there was no reverse causality (Supplementary Table S4). Funnel plots and scatter plots provide evidence of the symmetry and stability of SNPs (Supplementary Figures S3 and S4). Leave-one-out sensitivity analyses were performed to assess the robustness of the overall causality. The findings indicated that there were no statistically significant variations in the estimated effects when systematically excluding individual IVs and reiterating the MR analyses (Figure S5). This suggests that the observed effects cannot be solely attributed to any specific genetic instrument.

Since the urate data we used were non-sex-specific, the analysis procedure was replicated utilizing sex-specific GWAS statistics. The findings demonstrate a continued direct association between serum urate levels and female infertility, corroborating the initial outcomes (Supplementary Table S5).

### 3.2. Association between Serum Urate Levels and Cause-Specific Infertility

When analyzing cause-specific infertility, we observed a causal relationship between serum urate levels and female infertility due to cervical, vaginal, other, or unspecified origin (IVW ratio: OR = 1.16, 95% CI = 1.06, 1.26) (Table 1, Figure S1). However, no causal association was found for tubal and anovulatory types of infertility (Table 1, Figure S1). Cochran’s Q-tests did not detect heterogeneity among different causes of infertility (Table 1). Consistent results were observed through repeated analyses employing sex-specific data (Supplementary Table S4). MR-Egger analysis did not reveal any directional pleiotropy for the IVs, with intercepts of *p* = 0.28 for female infertility (tubal origin), intercepts of *p* = 0.33 for female infertility (anovulation), and intercept *p* = 0.54 for female infertility (cervical, vaginal, other, or unspecified origin) (Table 1). The MR-PRESSO test also shows no potential horizontal pleiotropy (Table 1). Funnel plots and scatter plots provide evidence of the symmetry and stability of SNPs (Supplementary Figures S3 and S4). A directionality test conducted by MR Steiger confirmed our estimation of potential causal direction (Supplementary Table S4). Leave-one-out sensitivity analyses were conducted to verify the robustness of the overall causality, and the results showed no significant differences in the estimated effects when systematically removing individual IVs and repeating the MR analyses (Figure S5), indicating that the observed effects cannot be attributed to any single genetic instrument.

### 3.3. Multivariate MR Analysis

Obesity is a potential confounder of female infertility. To account for potential horizontal pleiotropy via obesity, a multivariate MR analysis was conducted, adjusting for IV and potential confounder. After adjusting for BMI, the multivariate MR analysis conducted on uric acid levels revealed that BMI was not a significant risk factor for female infertility development (*p* = 0.320) (Table 2). However, uric acid exhibited a persistent positive and causal association with the risk of female infertility (IVW OR = 1.13, 95% CI: 1.04, 1.22, *p* = 0.03) and female infertility due to cervical, vaginal, other or unspecified origin (IVW OR = 1.12, 95% CI: 1.04, 1.22, *p* = 0.06) (Table 2).

## 4. Discussion

Excessive serum urate accumulation can lead to organ and system damage [42,43]. Substantial evidence supports the direct impact of urate levels on inflammation and abnormal lipid metabolism [20,44]. Reproductive diseases, such as adenomyosis, endometriosis, fibroids, and PCOS, as well as unexplained infertility, share inflammatory pathways and hormonal abnormalities, potentially reducing pregnancy success through a common process [45]. Previous epidemiological studies have reported elevated serum urate levels in patients with PCOS and endometriosis [46,47]. In one cross-sectional study, the latest nationally representative data from the 2013–2020 NHANES was utilized to confirm an association between elevated serum urate levels and infertility in women [29]. Furthermore, a dose-dependent increase in the strength of this association was observed [48]. A recent MR study had a similar finding of a causal relationship between high uric acid and increased risk of female infertility [49].

Our study represents the MR study investigating the causal relationship between multiple female infertility phenotypes and urate levels. Observational studies are often confounded by factors or reverse causality, issues that can be mitigated in MR studies. In this investigation, we screened a comprehensive GWAS database and identified 93 SNPs closely associated with both urate and infertility. We employed four complementary MR methods to analyze the causal relationship between the two, revealing a causal link between elevated urate and infertility. Notably, elevated urate levels were identified as a risk factor for the development of infertility in women (OR = 1.14, 95% CI = 1.04, 1.25). Additionally, we discovered that high urate levels increased the risk of female infertility from cervical, vaginal, other, or unspecified origins (OR = 1.16, 95% CI = 1.06, 1.26), although the precise mechanism by which elevated urate contributes to this remains unclear.

Several limitations should be acknowledged in this study. Firstly, the number of SNPs included in our analysis was limited, necessitating larger GWAS studies with more SNPs to enhance the robustness of MR investigations. Secondly, the effect size of most genetic variants was moderate due to the small variance explained by these identified SNPs. Consequently, larger sample sizes and more reliable instruments are required to accurately detect causal relationships between exposures and outcomes. Thirdly, our study exclusively focused on populations of European ancestry, precluding the assessment of potential genetic differences among various ethnic groups, countries, and regions.

Nonetheless, our study possesses several strengths. Firstly, we derived IVs for urate from the largest GWAS dataset available. Secondly, the MR approach allowed us to obtain a substantial amount of outcome genetic data from publicly accessible genetic datasets. Meta-analysis, which consolidates statistical data, proved as effective as collecting individual-level data from numerous small studies. Lastly, sample overlap was minimized by selecting samples from two distinct databases: female infertility samples from the Finnish database and urate samples from the CKDGen consortium.

In conclusion, our MR analyses revealed a causal effect of serum urate levels on female infertility, suggesting that elevated serum urate levels are indicative of an increased risk of infertility in European women.

## Figures and Tables

**Table 1:** MR results of the causal effect of urate on total and cause-specific female infertility.

Outcomes	No. of SNPs	Method	Beta (SE)	OR (95% CI)	*p* Value	Heterogeneity Test		Pleiotropy Test		
						Cochran’s Q (I2)	*p* Value	MR Egger		MR-Presso Global-Test
								Egger Intercept	*p* Value	*p* Value
Total female infertility	93	Inverse variance weighted	0.13 (0.05)	1.14 (1.04, 1.25)	0.009	112.45	0.063			
	93	MR Egger	0.18 (0.07)	1.25 (1.05, 1.36)	0.006	113.65	0.063	−0.004	0.33	0.061
	93	Weighted median	0.17 (0.06)	1.19 (1.04, 1.34)	0.005					
	93	Weighted model	0.16 (0.06)	1.17 (1.05, 1.31)	0.53					
Female infertility (tubal origin)	93	Inverse variance weighted	0.10 (0.11)	1.00 (0.80, 1.24)	0.98	87.31	0.59			
	93	MR Egger	0.04 (0.17)	1.14 (0.82, 1.57)	0.44	88.51	0.58	−0.001	0.28	0.057
	93	Weighted median	0.06 (0.17)	1.06 (0.76, 1.47)	0.73					
	93	Weighted model	0.03 (0.15)	1.01 (0.74, 1.37)	0.95					
Female infertility (anovulation)	93	Inverse variance weighted	0.09 (0.10)	1.10 (0.89, 1.35)	0.37	112.45	0.06			
	93	MR Egger	0.14 (0.15)	1.16 (0.86, 1.56)	0.34	113.65	0.06	−0.004	0.33	0.085
	93	Weighted median	0.10 (0.15)	1.11 (0.83, 1.49)	0.49					
	93	Weighted model	0.13 (0.12)	1.14 (0.89, 1.45)	0.30					
Female infertility (cervigal, vaginal, other, or unspecified origin)	93	Inverse variance weighted	0.15 (0.04)	1.16 (1.06, 1.26)	0.001	87.07	0.60			
	93	MR Egger	0.18 (0.06)	1.19 (1.05, 1.35)	0.007	87.46	0.61	−0.002	0.54	0.076
	93	Weighted median	0.18 (0.07)	1.19 (1.05, 1.35)	0.007					
	93	Weighted model	0.15 (0.06)	1.17 (1.04, 1.31)	0.008					

Abbreviation: MR, Mendelian randomization; SNPs, single nucleotide polymorphisms; IVW, inverse variance weighted; OR, odds ratio; CI, confidence interval.

**Table 2:** Multivariate MR results of the causal effect of urate on total and cause-specific female infertility.

Outcomes	Exposures	Beta	SE	OR (95% CI)	*p* Value
Total female infertility	Urate levels	0.120	0.040	1.13 (1.04, 1.22)	0.003
	Body mass index	−0.086	0.086	0.92 (0.78, 1.09)	0.32
Female infertility (tubal origin)	Urate levels	0.038	0.103	1.04 (0.85, 1.27)	0.71
	Body mass index	−0.076	0.219	0.93 (0.60, 1.43)	0.73
Female infertility (anovulation)	Urate levels	0.196	0.092	1.22 (1.02, 1.46)	0.06
	Body mass index	0.111	0.196	1.12 (0.76, 1.64)	0.57
Female infertility (cervical, vaginal, other or unspecified origin)	Urate levels	0.117	0.042	1.12 (1.04, 1.22)	0.006
	Body mass index	−0.164	0.091	0.85 (0.71, 1.02)	0.072

## Data Availability

The data underlying this article are available in the article and in its online Supplementary Material.

## Supplementary Materials

The following supporting information can be downloaded at the following link: https://www.mdpi.com/article/10.3390/metabo14100516/s1. Supplementary Table S1: Baseline characteristics of the study population; Supplementary Table S2: Characteristics of SNPs used as genetic instruments for urate in the present MR study; Supplementary Table S3: Genetic association of genetic variants with female infertility and its other phenotypes; Supplementary Table S4: Female-specific MR results of the causal effect of urate on total and cause-specific female infertility; Supplementary Table S5: Summary result of the MR Steiger; Supplementary Figure S1: Forest plot of the causal effects of urate associated SNPs on total and cause-specific female infertility; Supplementary Figure S2: Forest plots in the present Mendelian randomization study; Supplementary Figure S3: Funnel plots in the present Mendelian randomization study; Supplementary Figure S4: Scatter plots in the present Mendelian randomization study; and Supplementary Figure S5: Leave-one-out sensitivity analysis in the present Mendelian randomization study.
