# Supplementary material for: The Causal Effect of Urate Level on Female Infertility: A Mendelian Randomization Study
Source: Metabolites. 2024 Sep 25;14(10):516. doi: 10.3390/metabo14100516 (PMC11509567; doi:10.3390/metabo14100516)

Supplementary Table S1 Baseline characteristics of the study population

Traits	Consortium	Infertility Diagnostic Codes in ICD10	Year	Sample Size	Sex	N cases	N controls	number of SNPs tested	Population Studied
Urate	CKDGen Consortium	/	2018	288,649	Males and Females	NA	NA	8,217,338	European
Urate	GUGC	/	2015	4690	Females	NA	NA	2,521,972	European
Female infertility	FinnGen	N97	2023	120,706	Females	13,142	107,564	16,377,038	European
Female infertility, tubal origin	FinnGen	N97.100	2023	109,047	Females	1483	107564	16,379,641	European
Female infertility, associated with anovulation	FinnGen	N97.000	2023	110,005	Females	2441	107564	16,379,757	European
Female infertility, cervigal, vaginal, other or unspecified origin	FinnGen	N97.300, N97.801	2023	118,912	Females	11348	107564	16,379,641	European
BMI	MRC-IEU	/	2018	461,460	Males and Females	NA	NA	9851867	European

Supplementary Table S2 Characteristics of SNPs used as genetic instruments for urate in the present MR study (PMID: 31578528 for urate)

Exposure	SNP	Chr	Position	Nearest gene	EA	NEA	EAF	SNP-Exposure association			R^{2} [a]	F-statistic [b]	Linkage disequilibrium [c]	Proxy [d]
								Beta	SE	P value				
Urate	rs2480712	1	2156999	SKI	C	G	0.66	0.024	0.004	6.25E-09	0.0001	36		
Urate	rs4646068	1	15828704	CASP9	T	C	0.69	0.024	0.004	7.71E-09	0.0001	36		
Urate	rs79598313	1	27284913	KDF1	T	C	0.03	0.100	0.013	9.22E-15	0.0002	59		
Urate	rs141990161	1	119943525	HAO2	T	C	0.98	0.133	0.023	1.59E-08	0.0001	33		
Urate	rs10910845	1	145723120	NBPF20	A	C	0.47	0.058	0.004	1.50E-51	0.0007	210		
Urate	rs11204701	1	150662179	GOLPH3L	T	A	0.22	0.036	0.005	1.04E-14	0.0002	52	$r^2 > 0.001$	
Urate	rs2070803	1	155157715	TRIM46	A	G	0.58	0.053	0.004	4.09E-41	0.0006	176		
Urate	rs12134456	1	155722506	GON4L	G	C	0.37	0.043	0.005	7.61E-19	0.0003	74		
Urate	rs2760215	1	163675883	LOC100422212	C	T	0.50	0.025	0.004	5.81E-11	0.0001	39		
Urate	rs12037861	1	221038177	HLX-AS1	A	T	0.70	0.023	0.004	3.39E-08	0.0001	33		
Urate	rs2867112	2	651349	TMEM18	T	G	0.83	0.035	0.005	9.84E-12	0.0002	49		
Urate	rs72782806	2	15788511	DDX1	A	G	0.26	0.025	0.004	8.12E-09	0.0001	39		
Urate	rs72804854	2	27159850	DPYSL5	G	A	0.04	0.056	0.010	4.97E-08	0.0001	31	$r^2 > 0.001$	
Urate	rs1260326	2	27730940	GCKR	T	C	0.40	0.070	0.004	4.61E-69	0.0011	306		
Urate	rs62140395	2	28244926	BABAM2	C	G	0.12	0.052	0.007	3.17E-15	0.0002	55	$r^2 > 0.001$	
Urate	rs12472381	2	59321225	LINC01122	A	G	0.39	0.022	0.004	1.80E-08	0.0001	30		
Urate	rs12987661	2	69813458	AAK1	T	C	0.87	0.041	0.006	1.44E-12	0.0002	47		
Urate	rs759219	2	71163225	ATP6V1B1	C	T	0.56	0.022	0.004	7.93E-09	0.0001	30	$r^2 > 0.001$	
Urate	rs17050272	2	121306440	LINC01101	A	G	0.42	0.032	0.004	1.57E-15	0.0002	64		
Urate	rs11683692	2	145509615	TEX41	C	T	0.06	0.048	0.008	1.32E-08	0.0001	36		
Urate	rs1234413	2	148844369	MBD5	C	T	0.56	0.022	0.004	7.08E-09	0.0001	30		
Urate	rs9287911	2	170037294	LRP2	A	T	0.25	0.038	0.004	1.13E-17	0.0003	90		
Urate	rs187355703	2	176993583	HOXD8	G	C	0.03	0.086	0.013	2.70E-11	0.0002	44		
Urate	rs1047891	2	211540507	CPS1	C	A	0.69	0.024	0.004	2.09E-08	0.0001	36		
Urate	rs9288447	2	213083638	ERBB4	C	T	0.45	0.023	0.004	3.27E-09	0.0001	33		
Urate	rs699465	3	52310442	WDR82	A	G	0.14	0.045	0.006	6.45E-15	0.0002	56		

Urate	rs2581817	3	53071797	SFMBT1	C	G	0.42	0.048	0.004	4.87E-35	0.0005	144		Not available
Urate	rs11128111	3	69145632	ARL6IP5	C	T	0.52	0.021	0.004	4.64E-08	0.0001	28		
Urate	rs7640441	3	125118082	ZNF148	C	A	0.75	0.028	0.005	1.26E-09	0.0001	31		
Urate	rs11718633	3	126012421	KLF15	C	T	0.80	0.028	0.005	7.05E-09	0.0001	31	$r^2 > 0.001$	
Urate	rs80120242	3	132235344	DNAJC13	T	A	0.05	0.062	0.010	1.87E-09	0.0001	38	$r^2 > 0.001$	
Urate	rs62294340	3	169155476	MECOM	G	A	0.64	0.022	0.004	5.00E-08	0.0001	30		
Urate	rs2030869	4	9419763	DEFB131A	A	T	0.98	0.215	0.027	3.64E-15	0.0002	63	$r^2 > 0.001$	
Urate	rs4447862	4	9931645	SLC2A9	C	G	0.73	0.330	0.004	0.00E+00	0.0230	6806		
Urate	rs2108878	4	10527342	CLNK	T	C	0.70	0.107	0.004	7.54E-143	0.0025	716	$r^2 > 0.001$	
Urate	rs98270	4	48019323	NIPAL1	A	G	0.36	0.022	0.004	4.21E-08	0.0001	30		
Urate	rs10857147	4	81181072	FGF5	A	T	0.71	0.024	0.004	2.21E-08	0.0001	36		
Urate	rs116183010	4	88468158	SPARCL1	A	G	0.02	0.093	0.014	2.14E-11	0.0002	44	$r^2 > 0.001$	
Urate	rs74904971	4	89050026	ABCG2	A	C	0.11	0.254	0.006	0.00E+00	0.0062	1792		
Urate	rs2704571	4	89960285	FAM13A	A	G	0.32	0.023	0.004	1.35E-08	0.0001	33	$r^2 > 0.001$	
Urate	rs12644329	4	143634746	INPP4B	G	a	0.38	0.023	0.004	8.86E-09	0.0001	33		
Urate	rs1440411	4	144158285	USP38	C	T	0.43	0.028	0.004	1.08E-12	0.0002	49		
Urate	rs455213	5	34660235	RAI14	C	T	0.46	0.027	0.004	6.05E-12	0.0002	46		
Urate	rs10942549	5	72426137	TMEM171	G	C	0.69	0.042	0.004	1.64E-22	0.0004	110		
Urate	rs76004499	5	176705865	NSD1	G	C	0.03	0.074	0.013	3.26E-08	0.0001	32		
Urate	rs12530084	6	7214676	RREB1	T	C	0.22	0.066	0.005	9.55E-48	0.0006	174		
Urate	rs138739848	6	25250492	LOC101928663	T	C	0.05	0.062	0.009	1.30E-11	0.0002	47	$r^2 > 0.001$	
Urate	rs1359232	6	25809716	SLC17A1	C	A	0.53	0.091	0.004	4.12E-126	0.0018	518		
Urate	rs35942569	6	26339131	BTN3A2	G	A	0.08	0.067	0.007	3.51E-20	0.0003	92		
Urate	rs66975207	6	26942146	LINC00240	C	A	0.08	0.057	0.007	6.23E-15	0.0002	66		
Urate	rs35848276	6	27521096	ZNF184	T	C	0.09	0.053	0.007	8.92E-14	0.0002	57	$r^2 > 0.001$	
Urate	rs68188794	6	28080777	ZSCAN16-	C	T	0.08	0.059	0.008	2.72E-14	0.0002	54	$r^2 > 0.001$	

				AS1										
Urate	rs4713177	6	28673998	LINC00533	A	G	0.11	0.051	0.007	3.99E-13	0.0002	53		Not available
Urate	rs429479	6	29372323	OR12D2	G	A	0.10	0.048	0.007	2.23E-12	0.0002	47	$r^2 > 0.001$	
Urate	rs742493	6	40998167	UNC5CL	T	C	0.88	0.039	0.006	9.79E-11	0.0001	42	$r^2 > 0.001$	
Urate	rs1574430	6	43269029	SLC22A7	A	C	0.41	0.029	0.004	2.77E-14	0.0002	53	$r^2 > 0.001$	
Urate	rs10223666	6	43805502	VEGFA	C	G	0.70	0.046	0.004	6.62E-28	0.0005	132		
Urate	rs4897160	6	126223944	NCOA7	A	G	0.48	0.030	0.004	1.96E-14	0.0002	56		
Urate	rs62435145	7	1286567	UNCX	T	G	0.69	0.042	0.005	2.36E-16	0.0002	71		
Urate	rs13226650	7	73017005	MLXIPL	A	G	0.81	0.049	0.005	1.34E-23	0.0003	96		
Urate	rs11551890	7	97845713	TECPR1	A	G	0.51	0.023	0.004	2.40E-08	0.0001	33		
Urate	rs10480300	7	151406005	PRKAG2	T	C	0.28	0.030	0.004	4.26E-12	0.0002	56		
Urate	rs34861762	8	23748420	STC1	T	C	0.42	0.034	0.004	3.50E-19	0.0003	72		
Urate	rs2466077	8	32432753	NRG1	G	T	0.47	0.022	0.004	1.78E-08	0.0001	30	$r^2 > 0.001$	
Urate	rs2943539	8	76479839	HNF4G	T	C	0.48	0.041	0.004	6.42E-28	0.0004	105		
Urate	rs62517932	8	77031593	LINC01111	A	G	0.08	0.045	0.007	3.80E-10	0.0001	41	$r^2 > 0.001$	
Urate	rs10956924	8	95678312	ESRP1	C	T	0.72	0.024	0.004	1.79E-08	0.0001	36		
Urate	rs10971420	9	33125000	B4GALT1	T	C	0.69	0.031	0.004	4.14E-14	0.0002	60		
Urate	rs56106601	9	130770484	FAM102A	A	C	0.95	0.061	0.009	2.67E-11	0.0002	46		
Urate	rs74440730	10	16920892	CUBN	C	A	0.11	0.037	0.006	2.22E-09	0.0001	38		
Urate	rs10994860	10	52645424	A1CF	T	C	0.18	0.064	0.005	1.31E-36	0.0006	164	$r^2 > 0.001$	
Urate	rs1649078	10	60293320	BICC1	C	A	0.52	0.039	0.004	4.08E-19	0.0003	95		
Urate	rs1171617	10	61467182	SLC16A9	T	G	0.77	0.079	0.005	1.81E-66	0.0009	250		
Urate	rs9420446	10	88880689	FAM35A	C	T	0.86	0.038	0.006	1.13E-11	0.0001	40		
Urate	rs35198068	10	114754784	TCF7L2	T	C	0.71	0.025	0.004	5.85E-09	0.0001	39		
Urate	rs35506085	11	2165576	IGF2	G	A	0.81	0.029	0.005	1.50E-08	0.0001	34		
Urate	rs3925584	11	30760335	DCDC1	T	C	0.55	0.030	0.004	1.66E-15	0.0002	56		
Urate	rs71456318	11	64332862	SLC22A11	A	C	0.48	0.079	0.004	4.41E-92	0.0013	390		
Urate	rs10896028	11	65432187	RELA	T	A	0.35	0.048	0.004	4.10E-33	0.0005	144	r2 > 0.001	

Urate	rs3862387	11	65939921	PACS1	G	T	0.13	0.033	0.006	3.74E-09	0.0001	30	
Urate	rs10892354	11	119238381	USP2	T	C	0.38	0.030	0.004	2.60E-13	0.0002	56	
Urate	rs7303595	12	15359063	RERG	A	T	0.34	0.025	0.004	7.05E-10	0.0001	39	
Urate	rs7315236	12	52251933	LOC105369971	T	C	0.36	0.029	0.004	1.91E-13	0.0002	53	$r^2 > 0.001$
Urate	rs12313306	12	57751854	R3HDM2	C	T	0.75	0.076	0.004	6.74E-65	0.0012	361	
Urate	rs12368865	12	58422642	LINC02403	A	G	0.91	0.046	0.007	1.32E-11	0.0001	43	$r^2 > 0.001$
Urate	rs10774625	12	111910219	ATXN2	A	G	0.48	0.032	0.004	5.54E-17	0.0002	64	
Urate	rs17696736	12	112486818	NAA25	G	A	0.43	0.028	0.004	1.76E-12	0.0002	49	
Urate	rs1800574	12	121416864	HNF1A	C	T	0.97	0.081	0.012	2.84E-12	0.0002	46	
Urate	rs28530689	12	122500748	LOC100506691	A	C	0.51	0.032	0.004	1.27E-16	0.0002	64	
Urate	rs12423664	12	133069894	FBRSL1	A	G	0.15	0.042	0.006	1.75E-13	0.0002	49	
Urate	rs7986094	13	31029931	HMGB1	C	A	0.70	0.024	0.004	1.74E-08	0.0001	36	
Urate	rs626277	13	72347696	DACH1	A	C	0.59	0.026	0.004	2.69E-11	0.0001	42	
Urate	rs861536	14	104167564	KLC1	A	G	0.62	0.024	0.004	2.16E-09	0.0001	36	
Urate	rs1478604	15	39873321	THBS1	C	T	0.29	0.026	0.004	4.49E-10	0.0001	42	
Urate	rs2929508	15	72246964	MYO9A	T	A	0.74	0.029	0.005	3.65E-09	0.0001	34	$r^2 > 0.001$
Urate	rs8040109	15	73334225	NEO1	A	C	0.71	0.025	0.004	5.85E-09	0.0001	39	
Urate	rs2472297	15	75027880	CYP1A1	C	T	0.75	0.028	0.005	1.50E-08	0.0001	31	
Urate	rs10851885	15	76304503	NRG4	G	A	0.24	0.054	0.005	4.16E-32	0.0004	117	
Urate	rs157768	15	76833779	SCAPER	C	T	0.91	0.040	0.007	7.96E-09	0.0001	33	$r^2 > 0.001$
Urate	rs55781567	15	78857986	CHRNA5	C	G	0.65	0.023	0.004	1.10E-08	0.0001	33	$r^2 > 0.001$
Urate	rs12908437	15	99287375	IGF1R	T	C	0.38	0.046	0.004	1.56E-30	0.0005	132	
Urate	rs4997081	16	20365234	UMOD	G	C	0.80	0.030	0.005	4.18E-10	0.0001	36	
Urate	rs8050136	16	53816275	FTO	A	C	0.40	0.025	0.004	2.34E-10	0.0001	39	
Urate	rs62052820	16	69575238	MIR1538	A	G	0.21	0.041	0.005	2.81E-18	0.0002	67	
Urate	rs4788815	16	71634811	TAT	T	A	0.64	0.026	0.004	7.44E-11	0.0001	42	
Urate	rs9302635	16	72144174	DHX38	C	T	0.19	0.028	0.005	8.07E-09	0.0001	31	$r^2 > 0.001$

Urate	rs57652769	16	79753976	MAFTRR	C	T	0.69	0.036	0.004	8.56E-18	0.0003	81	
Urate	rs11644696	16	81572093	CMIP	A	G	0.48	0.022	0.004	1.41E-08	0.0001	30	
Urate	rs2453580	17	19438321	SLC47A1	T	C	0.60	0.025	0.004	7.01E-10	0.0001	39	
Urate	rs3794748	17	53365172	HLF	A	G	0.41	0.038	0.004	1.38E-21	0.0003	90	$r^2 > 0.001$
Urate	rs9895661	17	59456589	BCAS3	T	C	0.82	0.050	0.005	7.23E-23	0.0003	100	
Urate	rs11663816	18	57876227	MC4R	C	T	0.27	0.030	0.004	1.40E-12	0.0002	56	
Urate	rs57070985	19	4969053	KDM4B	A	G	0.65	0.029	0.004	2.04E-12	0.0002	53	$r^2 > 0.001$
Urate	rs10405423	19	7211311	INSR	A	C	0.66	0.039	0.004	1.07E-20	0.0003	95	
Urate	rs4808762	19	18326222	PDE4C	C	T	0.28	0.024	0.004	1.36E-08	0.0001	36	
Urate	rs2868194	19	33350060	SLC7A9	C	T	0.59	0.027	0.004	8.90E-12	0.0002	46	
Urate	rs35396326	19	45357003	NECTIN2	C	G	0.70	0.025	0.004	2.30E-08	0.0001	39	
Urate	rs62128132	19	50217955	CPT1C	C	T	0.03	0.118	0.015	1.32E-15	0.0002	62	
Urate	rs7267595	20	10643850	JAG1	A	C	0.51	0.023	0.004	3.15E-09	0.0001	33	
Urate	rs6119510	20	33287782	TP53INP2	G	T	0.40	0.023	0.004	3.20E-09	0.0001	33	
Urate	rs1800961	20	43042364	HNF4A	C	T	0.97	0.076	0.012	1.63E-10	0.0001	40	
Urate	rs219781	21	37832621	CLDN14	T	G	0.25	0.025	0.004	1.56E-08	0.0001	39	
Urate	rs12485100	22	44325516	PNPLA3	G	T	0.83	0.033	0.005	2.44E-10	0.0002	44	

Abbreviation: SNP, single nucleotide polymorphism; Chr, chromosome; EA, Effect allele; NEA, Non-effect allele; EAF, effect allele frequency; SE, standard error; OA, osteoarthritis.

a R2 was calculated using the following formula: (2×EAF×(1-EAF)×beta2)/[(2×EAF×(1-EAF)×beta2)+(2×EAF×(1-EAF)×N×SE2)], where EAF is the effect allele frequency, beta is the estimated effect on urate or gout, N is the sample size of the GWAS for the SNP-urate or gout association and SE is the standard error of the estimated effect.

b F statistic was calculated using the following formula: R2(N-2)/(1-R2), where R2 is the proportion of variance in urate or gout explained by each instrument and N is the sample size of the GWAS for the SNP-urate or gout association.

c SNPs with linkage disequilibrium (r2 > 0.001) were pruned.

d Proxy SNPs not available on the online platform SNiPA (https://snipa.helmholtz-muenchen.de/snipa3/) were removed.

Supplementary Table S3 Genetic association of genetic variants with Female infertility and its other phenotypes

SNP	Female infertility			Female infertility (tubal origin)			Female infertility (anovulation)			Female infertility (cervigal, vaginal, other or unspecified origin)		
	Beta	SE	P value	Beta	SE	P value	Beta	SE	P value	Beta	SE	P value
rs10223666	-0.157	0.441	0.723	-0.707	1.209	0.559	0.767	1.043	0.462	-0.104	0.461	0.821
rs10405423	0.067	0.503	0.894	0.833	1.379	0.546	-0.351	1.187	0.767	-0.062	0.526	0.907
rs10480300	0.637	0.783	0.416	0.610	2.150	0.777	2.310	1.860	0.214	0.687	0.820	0.402
rs10851885	0.194	0.494	0.694	-0.544	1.348	0.686	1.083	1.167	0.353	0.002	0.515	0.997
rs10857147	1.346	0.846	0.112	1.208	2.321	0.603	0.025	2.004	0.990	1.496	0.883	0.090
rs10892354	-0.317	0.633	0.617	-0.250	1.737	0.886	-0.603	1.500	0.688	0.117	0.663	0.860
rs10910845	-0.631	0.510	0.216	0.036	1.398	0.980	-2.579	1.202	0.032	-0.150	0.531	0.778
rs10942549	-0.700	0.863	0.417	-2.346	2.375	0.323	1.933	2.050	0.346	-0.913	0.900	0.311
rs10956924	-0.355	0.690	0.607	1.590	1.894	0.401	-0.174	1.639	0.915	-0.535	0.723	0.459
rs10971420	0.186	0.895	0.836	3.652	2.452	0.136	-0.848	2.114	0.688	0.229	0.933	0.807
rs11128111	-1.548	0.843	0.066	1.535	2.309	0.506	-2.596	1.996	0.193	-1.661	0.883	0.060
rs11551890	1.150	0.855	0.178	2.536	2.345	0.280	2.473	2.018	0.220	0.682	0.891	0.444
rs11683692	-0.833	0.754	0.269	-2.733	2.092	0.191	-1.525	1.788	0.394	0.050	0.790	0.950
rs1171617	0.120	0.324	0.711	0.863	0.886	0.330	0.241	0.766	0.753	0.080	0.338	0.813
rs12037861	-0.265	0.913	0.771	-3.009	2.509	0.230	-1.209	2.165	0.577	0.026	0.957	0.978
rs12313306	0.504	0.292	0.084	-0.674	0.796	0.397	0.858	0.689	0.213	0.597	0.304	0.049
rs1234413	-1.686	0.855	0.048	-1.918	2.345	0.413	-1.559	2.023	0.441	-1.364	0.891	0.126
rs12423664	0.721	0.669	0.281	3.786	1.833	0.039	0.921	1.581	0.560	0.552	0.698	0.428
rs12485100	0.315	0.327	0.336	2.048	1.979	0.301	0.067	1.703	0.969	-0.367	0.755	0.627
rs12530084	0.386	0.281	0.171	0.495	0.895	0.580	0.874	0.774	0.259	0.332	0.341	0.330
rs1260326	1.317	0.702	0.061	1.801	0.773	0.020	0.250	0.666	0.707	0.376	0.294	0.202
rs12987661	0.396	0.502	0.430	1.568	1.932	0.417	3.334	1.659	0.044	0.980	0.734	0.182
rs13226650	-0.104	0.214	0.626	0.031	1.371	0.982	-0.082	1.188	0.945	0.388	0.522	0.458
rs1359232	-0.297	0.631	0.638	-0.557	0.586	0.341	0.395	0.505	0.435	-0.143	0.223	0.522
rs141990161	0.350	0.686	0.610	1.255	1.723	0.466	-1.371	1.515	0.365	-0.391	0.660	0.554
rs1440411	0.015	0.869	0.986	0.996	1.882	0.597	-3.489	1.621	0.031	0.521	0.714	0.465

rs1478604	-0.605	0.485	0.212	5.585	2.388	0.019	0.415	2.065	0.841	-0.262	0.912	0.774
rs17050272	0.297	0.591	0.615	-1.197	1.326	0.366	-0.628	1.144	0.583	-0.562	0.505	0.266
rs1800574	-0.288	0.573	0.616	-0.500	1.622	0.758	1.856	1.397	0.184	-0.091	0.616	0.883
rs1800961	0.391	0.596	0.512	-0.644	1.565	0.681	-0.406	1.347	0.763	0.036	0.593	0.952
rs187355703	-0.494	0.707	0.485	0.412	1.632	0.801	2.524	1.421	0.076	0.172	0.621	0.781
rs2070803	0.202	0.355	0.569	0.484	1.943	0.803	-1.905	1.686	0.259	-0.036	0.744	0.961
rs219781	0.572	0.916	0.532	0.004	0.974	0.997	-0.642	0.840	0.445	0.209	0.370	0.571
rs2453580	-2.632	0.844	0.002	0.788	2.500	0.753	0.192	2.160	0.929	0.536	0.952	0.573
rs2480712	0.052	0.402	0.897	0.456	2.316	0.844	-3.740	1.996	0.061	-2.664	0.880	0.002
rs2760215	-0.760	0.760	0.317	1.131	1.104	0.306	-0.475	0.952	0.618	-0.017	0.421	0.968
rs2868194	0.337	0.704	0.632	-3.220	2.080	0.122	-0.568	1.792	0.751	-0.108	0.792	0.892
rs2943539	0.678	0.461	0.141	-0.385	1.930	0.842	2.744	1.659	0.098	-0.022	0.733	0.976
rs34861762	0.232	0.553	0.674	0.095	1.263	0.940	-0.483	1.088	0.657	0.598	0.480	0.214
rs35506085	-0.797	0.862	0.355	-0.176	1.518	0.907	1.432	1.306	0.273	0.135	0.576	0.814
rs3862387	0.542	0.712	0.446	0.714	2.359	0.762	-2.993	2.031	0.141	-0.297	0.897	0.741
rs3925584	-0.247	0.630	0.695	2.388	1.955	0.222	-0.876	1.685	0.603	0.515	0.745	0.490
rs4447862	0.174	0.065	0.007	-0.260	1.727	0.880	-1.307	1.487	0.379	0.050	0.657	0.939
rs455213	0.711	0.711	0.317	0.062	0.177	0.727	0.106	0.152	0.486	0.180	0.067	0.007
rs4646068	1.500	0.821	0.068	-0.100	1.952	0.959	2.526	1.681	0.133	0.778	0.744	0.296
rs4788815	-0.622	0.853	0.466	2.100	2.250	0.351	3.954	1.938	0.041	0.596	0.858	0.488
rs4897160	-0.054	0.762	0.944	-2.298	2.265	0.310	-3.618	2.041	0.076	-0.051	0.888	0.954
rs4997081	-0.033	0.627	0.958	1.981	2.088	0.343	-1.765	1.804	0.328	0.438	0.796	0.582
rs56106601	0.340	0.740	0.646	-0.160	1.717	0.926	-0.800	1.480	0.589	0.060	0.653	0.927
rs57652769	-0.456	1.033	0.659	0.123	2.030	0.952	1.250	1.753	0.476	0.413	0.773	0.593
rs6119510	-0.203	0.564	0.719	-2.670	2.759	0.333	1.454	2.390	0.543	0.259	1.062	0.807
rs62128132	-0.300	0.817	0.714	-2.583	1.550	0.096	-1.592	1.336	0.234	-0.083	0.589	0.887
rs62294340	0.675	0.534	0.206	-0.091	2.243	0.968	0.522	1.930	0.787	-0.326	0.852	0.702
rs62435145	1.941	0.955	0.042	3.047	1.446	0.035	0.451	1.242	0.717	0.195	0.550	0.723
rs626277	1.031	0.474	0.030	1.118	2.605	0.668	2.255	2.250	0.316	2.482	0.991	0.012

rs699465	1.031	0.738	0.163	-0.219	1.302	0.866	2.948	1.121	0.009	0.714	0.495	0.149
rs71456318	0.614	0.239	0.010	4.358	2.023	0.031	0.235	1.735	0.892	0.942	0.769	0.221
rs72782806	2.248	0.813	0.006	0.067	1.571	0.966	0.300	1.344	0.823	0.033	0.598	0.956
rs7303595	2.940	0.804	0.000	0.538	0.657	0.413	-0.463	0.567	0.414	0.468	0.251	0.062
rs74440730	0.559	0.924	0.545	3.287	2.243	0.143	-2.122	1.926	0.271	2.909	0.852	0.001
rs74904971	0.056	0.140	0.692	-2.644	2.384	0.267	1.028	2.056	0.617	-0.116	0.908	0.898
rs76004499	1.250	0.804	0.120	0.808	2.200	0.713	2.896	1.892	0.126	2.552	0.836	0.002
rs7640441	-0.450	0.739	0.543	-2.386	2.546	0.349	1.854	2.203	0.400	0.749	0.968	0.439
rs79598313	-0.557	0.509	0.274	-0.163	0.385	0.671	0.080	0.330	0.809	0.066	0.146	0.653
rs9287911	-0.016	0.539	0.977	0.255	2.201	0.908	2.236	1.895	0.238	1.635	0.841	0.052
rs9288447	0.278	0.835	0.739	-2.711	2.025	0.181	-0.686	1.750	0.695	-0.421	0.771	0.585
rs9420446	0.271	0.647	0.675	1.684	1.408	0.232	-0.200	1.196	0.867	-0.583	0.535	0.276
rs98270	-0.095	0.959	0.921	-1.163	1.482	0.432	1.716	1.274	0.178	-0.013	0.563	0.981
rs9895661	-0.524	0.482	0.277	0.052	2.287	0.982	-0.391	1.974	0.843	0.409	0.870	0.638

Supplementary Table S4 Summary result of the MR Steiger

Exposure	Outcome	Correct causal direction	P value
Urate	Female infertility	True	2.68E-25
Urate	Female infertility, tubal origin	True	1.34E-31
Urate	Female infertility, associated with anovulation	True	4.94E-19
Urate	Female infertility, cervigal, vaginal, other or unspecified origin	True	2.26E-16

Supplementary Table S5 Female-specific MR results of the causal effect of urate on total and cause-specific female infertility

.

Exposures	Outcomes	No. of SNPs	Method	Beta	SE	OR (95% CI)	P
Urate	Total Female infertility	3	MR Egger	0.090	0.091	1.094(0.905, 1.272)	0.505
		3	Weighted median	0.141	0.052	1.151(1.049, 1.252)	0.007
		3	Inverse variance weighted	0.149	0.048	1.160(1.065, 1.255	0.002
		3	Weighted mode	0.139	0.051	1.149(1.049, 1.249)	0.112
	Female infertility (tubal origin)	3	MR Egger	-0.230	0.214	0.795(0.375, 1.215)	0.478
		3	Weighted median	0.071	0.125	1.074(0.828, 1.310)	0.569
		3	Inverse variance weighted	0.134	0.173	1.143(0.804, 1.483)	0.439
		3	Weighted mode	0.071	0.120	1.074(0.838, 1.310)	0.613
	Female infertility (anovulation)	3	MR Egger	0.100	0.095	1.105(0.919, 1.291)	0.483
		3	Weighted median	0.145	0.051	1.156(1.055, 1.256)	0.005
		3	Inverse variance weighted	0.152	0.050	1.164(1.065, 1.263)	0.003
		3	Weighted mode	0.143	0.057	1.154(1.042, 1.265)	0.128
	Female infertility (cervigal, vaginal, other or unspecified origin)	3	MR Egger	-0.062	0.249	0.940(0.452, 1.428)	0.845
		3	Weighted median	0.034	0.131	1.035(0.779, 1.291)	0.793
		3	Inverse variance weighted	0.043	0.133	1.044(0.784, 1.304)	0.745
		3	Weighted mode	0.034	0.147	1.034(0.746, 1.322)	0.840

Abbreviation: MR, Mendelian randomization; SNPs, single nucleotide polymorphisms; IVW, inverse variance weighted; OR, odds ratio; CI, confidence interval

Supplementary Figure 1 Forest plot of the causal effects of urate associated SNPs on total and cause-specific female infertility

Outcome	Nsnp	Method	OR(95%CI)	P val
Total Female infertility	93	Inverse variance weighted	1.14(1.04,1.25)	0.009
	93	MR Egger	1.25(1.05,1.36)	0.006
	93	Weighted median	1.19(1.04,1.34)	0.005
	93	Weighted model	1.17(1.05,1.31)	0.53
Female infertility (tubal origin)	93	Inverse variance weighted	1.00(0.80,1.24)	0.98
	93	MR Egger	1.14(0.82,1.57)	0.44
	93	Weighted median	1.06(0.76,1.47)	0.73
	93	Weighted model	1.01(0.74,1.37)	0.95
Female infertility (anovulation)	93	Inverse variance weighted	1.10(0.89,1.35)	0.37
	93	MR Egger	1.16(0.86,1.56)	0.34
	93	Weighted median	1.11(0.83,1.49)	0.49
	93	Weighted model	1.14(0.89,1.45)	0.30
Female infertility (cervigal, vaginal, other or unspecified origin)	93	Inverse variance weighted	1.16(1.06,1.26)	0.001
	93	MR Egger	1.19(1.05,1.35)	0.007
	93	Weighted median	1.19(1.05,1.35)	0.007
	93	Weighted model	1.17(1.04,1.31)	0.008

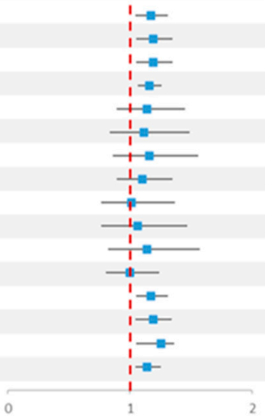

Supplementary Figure 2 Forest plots in the present Mendelian randomization study

(A) Forest plot of the causal effects of urate associated SNPs on female infertility

(B) Forest plot of the causal effects of urate associated SNPs on female infertility (tubal origin)

(C) Forest plot of the causal effects of urate associated SNPs on female infertility(anovulation)

(D) Forest plot of the causal effects of urate associated SNPs on female infertility (cervigal, vaginal, other or unspecified origin)

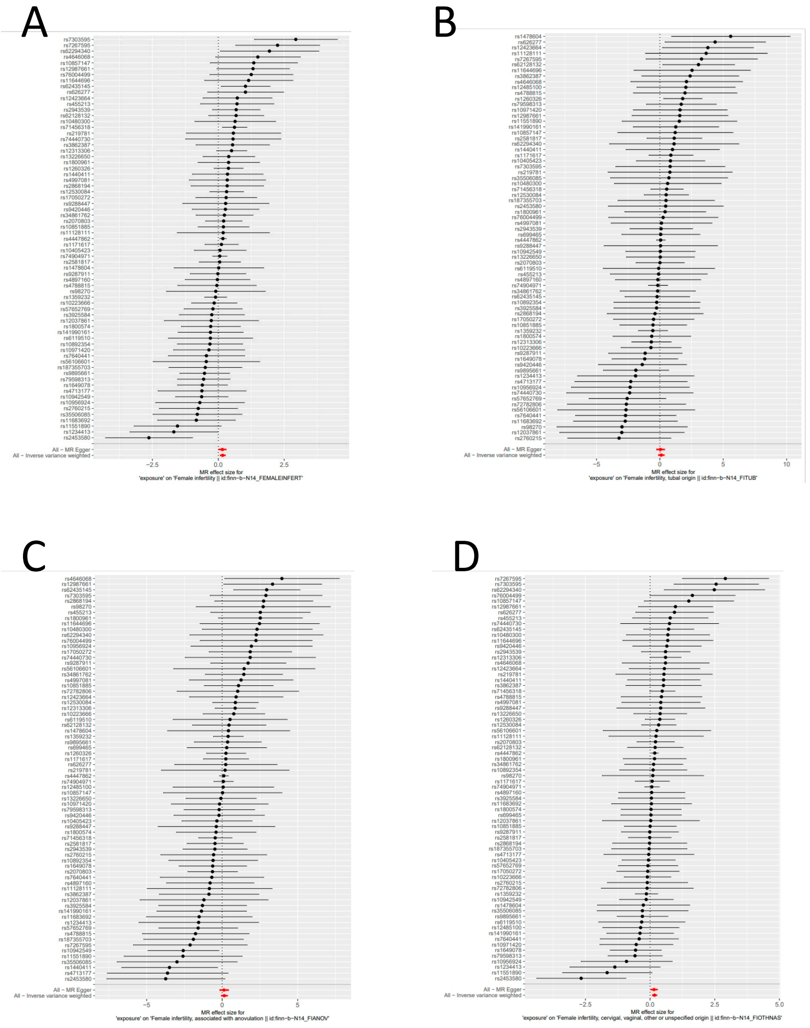

Supplementary Figure 3 Funnel plots in the present Mendelian randomization study

(A) Funnel plot of the causal effects of urate associated SNPs on female infertility

(B) Funnel plot of the causal effects of urate associated SNPs on female infertility (tubal origin)

(C) Funnel plot of the causal effects of urate associated SNPs on female infertility(anovulation)

(D) Funnel plot of the causal effects of urate associated SNPs on female infertility (cervigal, vaginal, other or unspecified origin)

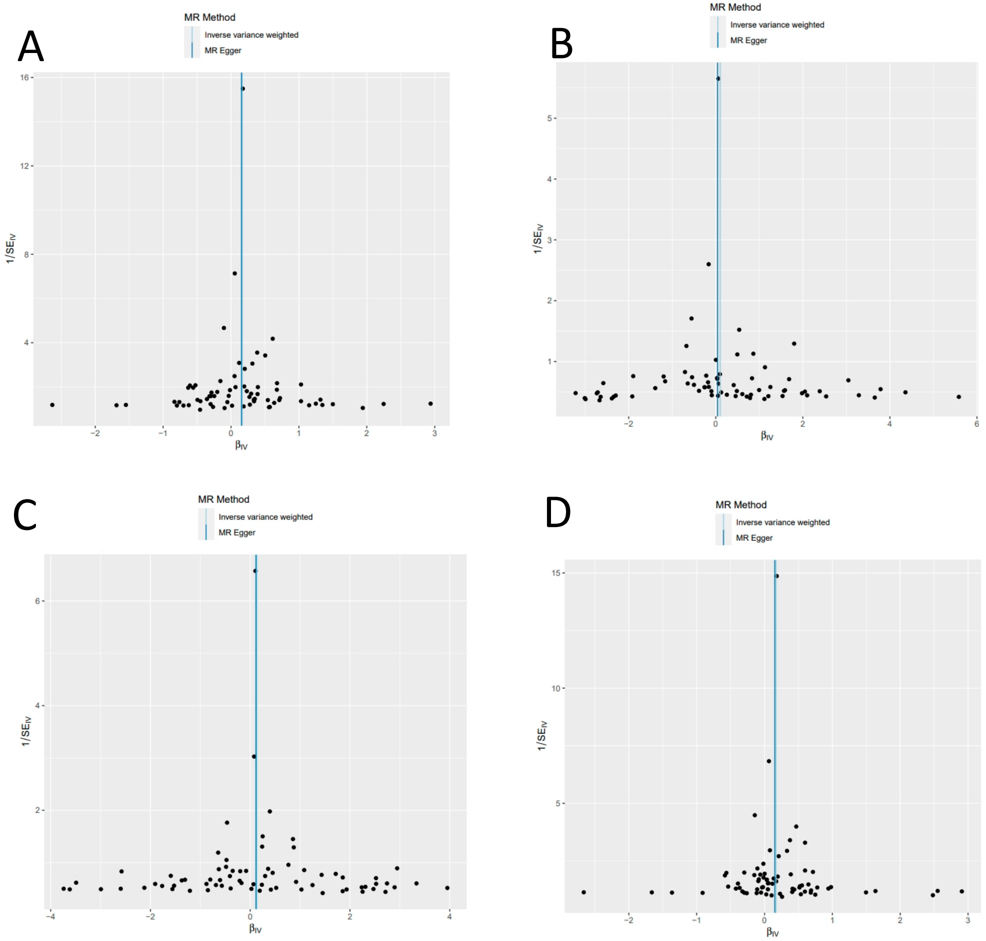

Supplementary Figure 4 Scatter plots in the present Mendelian randomization study

(A) Scatter plot of the causal effects of urate associated SNPs on female infertility

(B) Scatter plot of the causal effects of urate associated SNPs on female infertility (tubal origin)

(C) Scatter plot of the causal effects of urate associated SNPs on female infertility(anovulation)

(D) Scatter plot of the causal effects of urate associated SNPs on female infertility (cervigal, vaginal, other or unspecified origin)

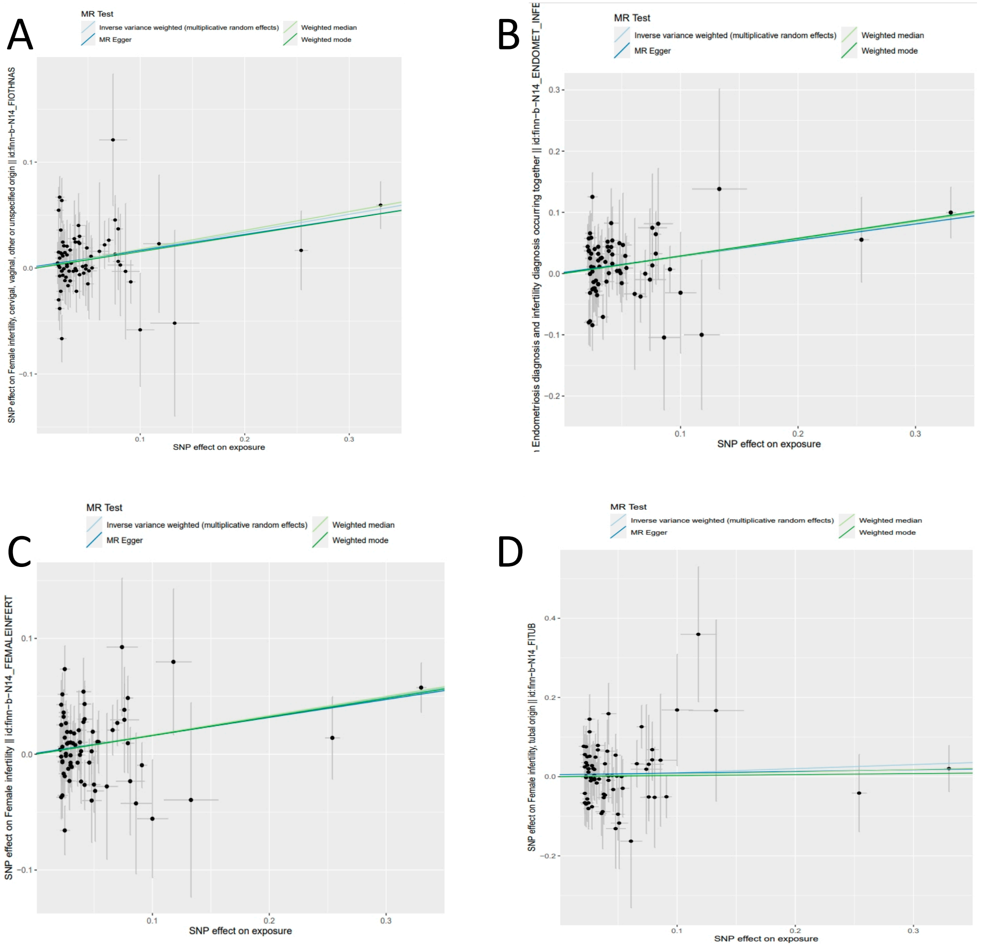

Supplementary Figure 5 Leave-one-out sensitivity analysis in the present Mendelian randomization study

(A) Leave-one-out sensitivity analysis of urate associated SNPs on female infertility

(B) Leave-one-out sensitivity analysis of urate associated SNPs on female infertility (tubal origin)

(C) Leave-one-out sensitivity analysis of urate associated SNPs on female infertility(anovulation)

(D) Leave-one-out sensitivity analysis of urate associated SNPs on female infertility (cervigal, vaginal, other or unspecified origin)

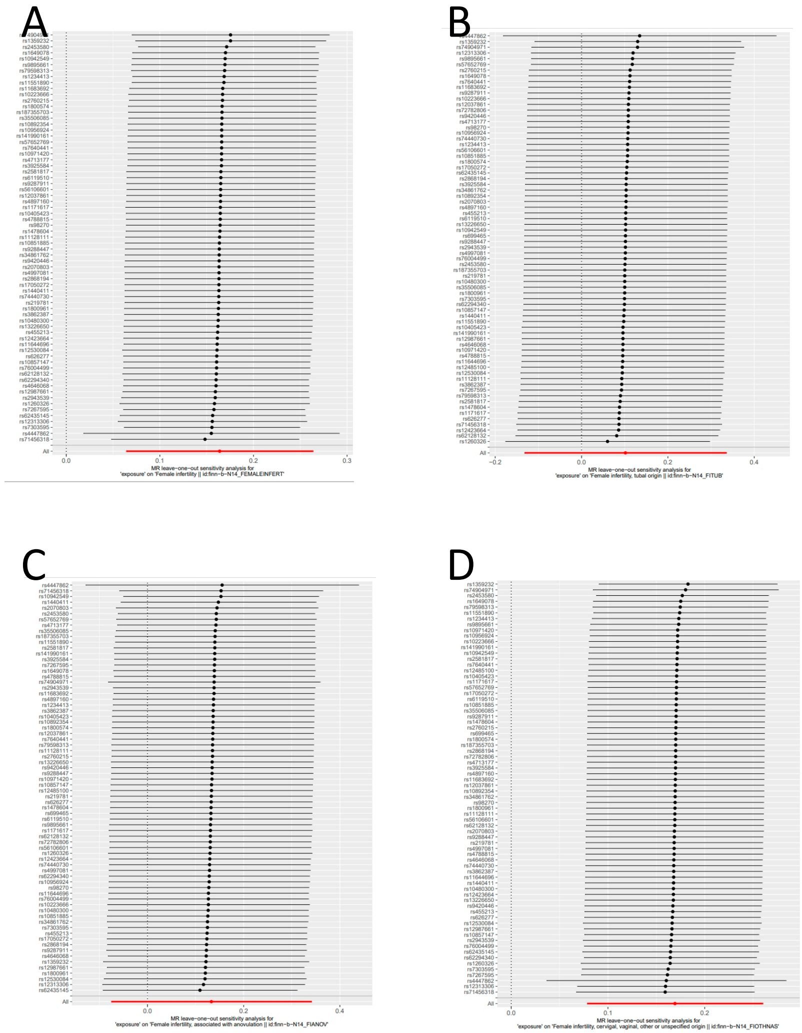

Supplement: Supplementary file 1 [file metabolites-14-00516-s001.zip › metabolites-3189932-supplementary.pdf]
